# Life events and donor lapse among blood donors in Denmark

**DOI:** 10.1111/vox.12842

**Published:** 2019-10-01

**Authors:** Tjeerd W. Piersma, Eva‐Maria Merz, René Bekkers, Wim de Kort, Steffen Andersen, Henrik Hjalgrim, Klaus Rostgaard, Kaspar René Nielsen, Henrik Ullum

**Affiliations:** ^1^ Department of Donor Medicine Research Sanquin Research Amsterdam the Netherlands; ^2^ Center for Philanthropic Studies Vrije Universiteit Amsterdam the Netherlands; ^3^ Department of Sociology Vrije Universiteit Amsterdam the Netherlands; ^4^ Department of Clinical Immunology, Copenhagen University Hospital Rigshospitalet Copenhagen Denmark; ^5^ Department of Social Medicine Academic Medical Center Amsterdam the Netherlands; ^6^ Department of Finance Copenhagen Business School Frederiksberg Denmark; ^7^ Department of Epidemiology Research Statens Serum Institut Copenhagen Denmark; ^8^ Department of Clinical Immunology Aalborg University Hospital Aalborg Denmark

**Keywords:** blood donors, donor lapse, life events, register data, replication

## Abstract

**Background and objectives:**

The likelihood of donating blood changes over the life course, with life events shown to influence entry to and exit from the donor population. While these previous findings provide valuable insights for donor management, blood collection agencies need to be cautious about generalizing findings to other countries as blood donor behaviour is context‐specific. To examine cross‐country variations in donor behaviour, the repeatability of a previous Dutch study on life events and blood donor lapse is examined by using a sample of Danish donors.

**Materials and methods:**

Register data from Statistics Denmark was linked to the Scandinavian Donations and Transfusions database (*n* = 152 887). Logistic regressions were conducted to examine the association between life events in 2009–2012 and blood donor lapse in 2013–2014.

**Results:**

Of the total sample, 69 079 (45·2%) donors lapsed. Childbirth and losing a job increased the lapsing risk by 11% and 16%, respectively, while health‐related events in the family (i.e. blood transfusion, disease and death) decreased the lapsing risk by 5%, 7% and 9%, respectively.

**Conclusion:**

Life events are associated with donor lapse of Danish donors. These results are comparable to previous findings from the Netherlands (i.e. childbirth and labour market transitions increased lapsing risk; health‐related events decreased lapsing risk), with two thirds of the associations being in the same direction. Differences between study results were mainly related to effect sizes and demographic compositions of the donor pools. We argue contextual factors to be of importance in blood donor studies.

## Introduction

Why and when do people give blood? Life events related to family composition, health of family and friends, and labour market transitions affect blood donor behaviour over the life course 1. Longitudinal studies from Germany and the Netherlands showed such life events to impact both *entry to* and *exit from* the donor pool, hereby illustrating the importance of longitudinal data to examine behavioural change across the blood donor career.

Regarding *entry to* the donor pool, German data showed that people who recently divorced or finished their education were more likely to start donating blood, while people who got a child or experienced the death of a parent were less likely to start a donor career Soliman et al.^2^ Regarding *exit from* the donor pool, Dutch data showed that childbirth, getting a job and losing a job increased the likelihood of donor lapse, while a blood transfusion for someone close and the death of a loved one decreased this likelihood 1.

Blood donor studies based on self‐reports indicated that barriers such as time constraints and decreased social connections are possible reasons to stop donating blood after experiencing a life event 3. Individuals with more available time, and more human and social capital (e.g. available health and social connections) were indeed more likely to donate blood 4, and these mechanisms were found to partially explain the association between life events and donor lapse among Dutch donors 1.

While these findings provide valuable insights for donor recruitment and retention, blood collection agencies (BCAs) need to be cautious in generalizing these findings to other countries. Blood donation and its antecedents are shown to be context‐specific, with contextual factors such as collection practices and cultural differences influencing blood donor attitudes and behaviour 5, 6. For instance, organizational variation between European BCAs has been shown to be related to donor diversity and loyalty 7. State‐run BCAs recruit more male donors of higher socio‐economic status who are likely to donate only once or twice, while BCAs under a Red Cross regime attract fewer but more loyal donors who are more equally distributed across socio‐economic groups 7.

To contribute to these country studies and identify more universal and possible context‐specific mechanisms for blood donor behaviour, we compared the association between life events and blood donor lapse between Denmark and the Netherlands by testing the repeatability 8 of previous findings 1 among a large sample of Danish donors. Moreover, by using register data from Statistics Denmark (SD) 9, linked to the Scandinavian Donations and Transfusions (SCANDAT) database 10, we were able to provide more accurate estimates of true effect sizes compared to findings from the Dutch survey study, for example by eliminating the possibility of false‐positive self‐reports as a result of recall bias.

## Materials and methods

In order to test the repeatability of previous findings, we selected data, procedures, measures and statistical analyses based on methodological decisions from the previous study (Appendix A and https://osf.io/9chtq/).

### Data and procedure

SD 9 and SCANDAT 10 were used to examine the association between life events and blood donor lapse among Danish donors. SD contains a wide array of information on the Danish population, ranging from societal information on geography, environment and economy to individual information on labour, income and wealth. SCANDAT contains data on all Danish blood donors and recipients who have been registered since the start of the computerized blood bank system in 1981 and has been used to study a variety of topics concerning blood donor behaviour and transfusion medicine because of the possibility to link SCANDAT to SD using Civil Registration Numbers (CRNs) as personal identifiers 10, 11. According to Danish law, register‐based research does not require ethical approval 12. For this study, we used CRNs to link SCANDAT to subsets of SD to examine if active blood donors – who made at least one donation in 2008 – experienced a life event in 2009‐2012 (i.e. health‐related event in the family, childbirth, losing a job, starting a job), and if these donors lapsed in 2013–2014.

From the sample of still active donors, those who were ineligible for future blood donations as a result of various medical (i.e. death, permanent deferral) and non‐medical reasons (i.e. migration, reaching the upper age limit of 67), were excluded from the sample. To retrieve this information, we used the ineligibility criteria list from the Danish blood bank 13 and checked whether any of these criteria applied to the donor using migration, death and hospitalization records from SD. Although these records do not contain information on all possible ineligibility reasons (e.g. temporary deferral related to malaria risk travel), it does capture the most prominent reasons for long‐term and permanent donor deferral relevant to our study design. The final sample consisted of 152 887 blood donors.

### Measures

#### Blood donor lapse

Blood donor lapse was defined as not making a donation for 24 consecutive months 14, with individual donation information retrieved from SCANDAT (0 = no donor lapse, 1 = donor lapse).

#### Life events

Inclusion of the six life events of interest was based on the previous analyses among blood donors from the Netherlands. Information on the occurrence of health‐related events among family members was retrieved from SCANDAT (i.e. blood transfusion) and SD (i.e. serious disease, death). Based on CRNs, we matched the donor to parents, children and siblings and then matched these family members to transfusion, hospitalization and death records. Using this information, we created a time‐dependent dichotomous variable representing whether the donor experienced a health‐related event in the family (e.g. 0 = no family member died, 1 = family member died). Information on childbirth was extracted from SD by matching newborn children to their parents using CRNs. We transformed these data such that the individual record of the donor included the CRN of their newborn child and recoded this as a time‐dependent dichotomous variable representing whether a child was born to the donor (0 = no childbirth, 1 = childbirth). Information on donors losing or starting a job was retrieved from SD. Time‐dependent dichotomous variables were created by tracking changes in employment status: from having a paid job to unemployment (0 = stayed at their job, 1 = lost their job), and from unemployment to having a paid job (0 = remained unemployed, 1 = started a job).

#### Mechanisms

To further explore the association between life events and donor lapse, we included two possible mediating mechanisms in the models. The total number of weekly working hours was included as a proxy for the available time to donate, with a higher number of weekly working hours representing higher costs to donate blood as time becomes a scarcer resource when working hours increase. Having other blood donors in the family was included as a proxy for the influence of social networks. Based on CRNs, we matched the donors to their parents, children, siblings and spouse and then matched the CRNs of these family members to SCANDAT to create a dichotomous variable representing whether the donor knew donors in the family (0 = does not know other donors, 1 = knows other donors).

#### Control variables

Sex, age, educational level and total number of previous blood donations were added to the model as control variables. Educational level was coded as lower, medium and higher education according to the International Standard Classification of Education (ISCED) 2011 15, a comprehensive framework to categorize educational levels and facilitate cross‐country comparisons of educational systems.

### Statistical analyses

Analyses were conducted using Stata 15 (College Station, TX: StataCorp LLC). Conducting logistic regression analyses, we estimated the association between life events and donor lapse. Donor inclusion requirements included having been ‘at risk’ for experiencing a life event of interest, that is women aged ≤45 and men aged ≤55 for childbirth (*n* = 117 266), unemployed donors for starting a job (*n* = 7570), employed donors for losing a job (*n* = 129 836), and all donors for the health‐related events (*n* = 152 887). We then obtained predicted probabilities for donors who were at risk for the event and did or did not experience the event, keeping the other variables in the model at their means.

As to examine whether childbirth differently affected donor lapse for men and women, we added an interaction term to the model. Mediation analyses (i.e. *Z*_Mediation_) 16 were performed as to test to what extent the costs of donating blood (i.e. increased working hours) and influences from the social network (i.e. knowing other blood donors) were able to explain the association between life events and donor lapse, only when this association proved to be statistically significant.

## Results

### Life events and the Danish donor population

Of the total donor sample, 45·2% (*n* = 69 079) lapsed in 2013–2014. Life events occurring most often to the donors were a serious disease in a family member (22·7%, *n* = 34 756), childbirth (15·1%, *n* = 23 056) and a blood transfusion for a family member (9·3%, *n* = 14 165). Table 1 shows descriptive statistics of all study measures. Compared to the Dutch donor sample (Appendix B), the Danish donor sample had a lower mean age (39·7 vs. 46·7), comprised of more male donors (53·1% vs. 47·2%), and had a lower average number of previous donations (11 vs. 21). Most notably, the number of lapsed donors was higher in Denmark than in the Netherlands (45·2% vs. 25·3%). With regard to the occurrence of health‐related life events, we noticed some proportional differences between the samples. A serious disease in a family member and death of a family member was reported by 40·5% and 18·9% of the Dutch donors and only observed among 22·7% and 8·8% of the Danish donors, respectively.

**Table 1:** Descriptive statistics of study measures, for total sample and stratified by donor status^a^

Characteristic	All donors (*n* = 152 887)	Active donors (*n* = 83 808; 54·8%)	Lapsed donors (*n* = 69 079; 45·2%)
Life events			
Transfusion	14 165 (9·3%)	8141 (9·7%)	6024 (8·7%)
Serious disease	34 756 (22·7%)	20 076 (24·0%)	14 680 (21·3%)
Death	13 381 (8·8%)	7955 (9·5%)	5426 (7·9%)
Childbirth	23 056 (15·1%)	11 584 (13·8%)	11 472 (16·6%)
Starting a job	5378 (3·5%)	2466 (1·2%)	2912 (4·2%)
Losing a job	6203 (4·1%)	2881 (3·4%)	3322 (4·8%)
Costs			
Working hours	22·6 (±8·23)	23·1 (±7 75)	21·8 (±8·76)
Social network			
Know other donors	40 067 (26·2%)	24 491 (29·2%)	15 576 (22·6%)
Age	39·7 (±12·30)	40·7 (±11·41)	38·1 (±13·28)
Sex			
Male	81 131 (53·1%)	47 126 (56·2%)	34 005 (49·2%)
Female	71 756 (46·9%)	36 682 (43·8%)	35 074 (50·8%)
Educational level			
Low	21 732 (14·2%)	10 969 (13·9%)	10 763 (15·6%)
Middle	87 448 (57·2%)	48 815 (58·3%)	38 633 (55·9%)
High	38 593 (25·4%)	21 613 (25·8%)	16 980 (24·6%)
Previous blood donations	11 (5–22)	13 (6–24)	9 (4–19)

a Data reported as mean (±SD), number (%) or median (25th–75th).

### Health of family members

#### Blood transfusion

Donors experiencing a blood transfusion for a family member were 5% less likely to lapse than donors who did not experience such an event (OR (95% CI) = 0·91 (0·88–0·94), *P* < 0·001). We found no evidence of a mediating role of knowing other blood donors in the association between a blood transfusion and donor lapse (Table 2). These results are comparable to earlier findings in the Netherlands, with Dutch donors experiencing a blood transfusion being 11% less likely to lapse.

**Table 2:** Results for the logistic regression analyses of life events on donor lapse^a^, mediated by the hypothesized mechanisms^b^

Life events & mechanisms^c^	Model A			Model B			*Z*_Mediation_	
	B^d^	SE	OR^e^ (95% CI)	B	SE	OR (95% CI)	% mediated^f^	*Z*‐value
Transfusion	−0·10***	0·02	0·91 (0·88–0·94)	−0·08***	0·02	0·91 (0·88–0·94)		
Know other donors				−0·40***	0·01	0·69 (0·68–0·71)	‐	0·05
Serious disease	−0·13***	0·01	0·88 (0·86–0·90)	−0·13***	0·01	0·88 (0·62–0·90)		
Know other donors				−0·37***	0·01	0·69 (0·67–0·71)	‐	0·01
Death	−0·17***	0·02	0·84 (0·81–0·87)	−0·17***	0·02	0·84 (0·81–0·87)		
Know other donors				−0·37***	0·01	0·69 (0·68–0·71)	‐	1·32
Childbirth	0·19***	0·02	1·21 (1·18–1·25)	‐	‐	‐	‐	‐
Start job	−0·02	0·05	0·98 (0·88–1·10)	‐	‐	‐	‐	‐
Lose job	0·28***	0·03	1·32 (1·25–1·39)	0·27***	0·03	1·31 (1·24–1·38)		
Know other donors				−0·36**	0·01	0·70 (0·68–0·72)	3%	5·36***

a Blood donor lapse for non‐medical reasons.

b Effects are estimated separately for each life event and its hypothesized mechanisms, and only when the donor is at risk for experiencing the life event: transfusion, disease and death (*n* = 152 887), childbirth (*n* = 117 266), start job (*n* = 7570) and lose job (*n* = 129 836).

c Results adjusted for donors' sex, age, educational level and the total number of previous blood donations.

d Estimated unstandardized regression coefficients.

e OR indicates the likelihood for donor lapse compared with the reference category.

f Percentage reported only when all paths in the model were significant 16.

*** *p* < 0·001, ^**^ *p* < 0·01 ^*^ *p* < 0·05 (two‐tailed tests).

#### Serious disease

Donors experiencing a serious disease in the family were 7% less likely to lapse than donors who did not experience a serious disease in the family (OR (95% CI) = 0·88 (0·86–0·90), *P* < 0·001). Subsequent mediation analysis showed no evidence of knowing other donors as an explaining mechanism for the association between a serious disease and donor lapse. These results were different from previous findings, where no significant difference was found among Dutch donors in their lapsing risk after a serious disease in the family.

#### Death

Donors having lost one of their family members were 9% less likely to lapse than donors who did not experience a family member's death (OR (95% CI) = 0.84 (0·81–0·87), *P* < 0·001). As was the case in the other health‐related events, further analysis revealed no evidence of knowing other donors being a mediating factor. The association between a family member's death and donor lapse is comparable to the one found among Dutch donors (i.e. an 8% increase in lapsing risk).

### Interrelationship between transfusion, disease and death

Since health‐related events were correlated to each other (Appendix C), with people receiving a blood transfusion being more likely to suffer from a serious disease and having a higher mortality risk, we explored their interrelationship in the negative association with blood donor lapse. Table 3 shows how the unstandardized coefficients changed when the health‐related events were introduced in a stepwise manner. While the coefficients for a blood transfusion halved after adding serious disease or a family member's death to the model, the coefficients of the latter two changed only slightly compared to their separate association with donor lapse. The association between a blood transfusion and lapse therefore seems to be subordinate to the association for a serious disease or death. When all three health‐related events were added to the model (Model 7), the association between a blood transfusion and lapse disappeared, while the coefficients for serious disease and death only slightly decreased. In this full model, death showed the strongest negative association with donor lapse, while serious disease showed the most robust association across the combined models. Comparing these results to the Dutch data, a different pattern emerged. Across all models, the relationships between blood transfusion and death with donor lapse were stable and significant, while no association was found between a serious disease and donor lapse (Appendix B).

**Table 3:** Results for the stepwise regression analyses^a^ of the health‐related life events on donor lapse^b^

Life events^c^	Model 1		Model 2		Model 3		Model 4		Model 5		Model 6		Model 7	
	B^d^	SE	B	SE	B	SE	B	SE	B	SE	B	SE	B	SE
Transfusion	−0·097***	0·012					−0·047*	0·019	−0·044*	0·019			−0·018	0·020
Serious disease			−0·128***	0·012			−0·115***	0·013			−0·097***	0·013	−0·095***	0·014
Death					−0·171***	0·019			−0·155***	0·020	−0·120***	0·019	−0·115***	0·021

a Effects are estimated separately for each health‐related life event in Models 1–3, in combination with one of the other health‐related life events in Models 4–6, simultaneously for all three health‐related life events in Model 7, and only when the donor is at risk for experiencing the event (*n* = 152 887).

b Blood donor lapse for non‐medical reasons.

c Results adjusted for donors' sex, age, educational level and the total number of previous blood donations.

d Estimated unstandardized regression coefficients.

e ***p < 0·001. **p < 0·01. *p < 0·05 (two‐tailed tests).

### Childbirth

Donors who got a child were 11% more likely to lapse than donors who did not get a child within the same period (OR (95% CI) = 1.21 (1·18–1·25), *P* < 0·001). While childbirth increased the lapsing risk for both men (OR (95% CI) = 1·18 (1·13–1·23), *P* < 0·001) and women (OR (95% CI) = 1·24 (1·18–1·29), *p* < 0·001), this association was significantly stronger for women than men. Compared to the study from the Netherlands, effect sizes differ considerably with Dutch donors who got a child being 56% more likely to lapse than donors who did not get a child. Moreover, in the Netherlands no significant difference was found between men and women in their lapsing risk after childbirth.

### Labour market transitions

#### Starting a job

No difference was found between donors who started a job and donors who stayed unemployed in their subsequent lapsing risk. In the absence of this main effect, we did not perform further mediation analyses and the role of increased working hours in donor lapse. These results differ quite strongly from earlier results in the Netherlands. Starting a job was positively associated with lapse among Dutch donors, with donors who started a job being 22% more likely to lapse compared with donors who stayed unemployed.

#### Losing a job

Donors who lost their job were 16% more likely to lapse than donors who stayed at their job during the same period (OR (95% CI) = 1·32 (1·25–1·39), *P* < 0·001). Mediation analysis showed that knowing fewer other blood donors was a significant mediator in the model, yet explaining only 3% of the variance in the relation between losing a job and donor lapse (z = 5·36, *P* < 0·001). These results repeat previous results from the Netherlands, but effect sizes differ slightly as Dutch donors were 35% more likely to lapse after losing their job, while the same effect was found for the small mediating role of knowing other donors in explaining the association between losing a job and donor lapse.

## Discussion

### Life events and donor lapse in Denmark

Using longitudinal data from SCANDAT and SD, we conclude that life events related to childbirth and work status, as well as health‐related events in the family, are associated with blood donor lapse. Childbirth and losing a job increased the risk of donor lapse, with childbirth being more detrimental for future blood donations of women than men. In contrast, health‐related events in the family decreased the risk of donor lapse. Once more, the likelihood to donate blood was shown to vary across the donor career, thus illustrating the dynamic nature of blood donor behaviour.

For a large part, these results are in line with previous findings from cross‐sectional blood donor studies based on self‐reports. Time constraints due to childbirth and family responsibilities appear to be among the main reasons for donors to discontinue donating blood 3, while health‐related events in the family were mentioned being a motivational factor to start donating blood 17 as well as a reason to continue donating over time 18. There were slight differences in the statistical influence of various health‐related events in the family with transfusion having a stronger effect in the Dutch dataset, but serious disease and death in the Danish. The interpretation of these highly correlated events is difficult, but we can robustly conclude that disease, transfusion and death in the family are motivational factors in donor retention. Our finding that people are more likely to lapse after losing their job aligns with previous research suggesting that social connections influence donation decisions 17. Although these connections did not play a mediating role in the associations between health‐related events and donor lapse, social connections in itself seem to impact on donor behaviour, as knowing fewer other donors was related to an increasing lapsing risk.

We also found some discrepancies compared with previous studies. Time constraints due to work are a common self‐reported reason for donors to stop donating blood 19, but we found no statistically significant association between starting a job and donor lapse. Also, we found no evidence for social connections explaining the association between health‐related events and donor lapse. Further understanding of underlying motivational mechanisms is important to provide insight in preventing donors from lapse at certain life stages.

### Country comparisons between Denmark and the Netherlands

We found the associations to be comparable between Denmark and the Netherlands. Of the events studied here, two thirds showed the same associative directions with donor lapse, while none of them showed opposite directions. Moreover, half of all confidence intervals showed an overlap between the studies. One might assume that certain (motivational) mechanisms are universal factors associated with blood donor behaviour across the donor career in Western high‐income countries. Childbirth constraints a person's available time 20, losing a job decreases self‐perceived health 21, and health‐related events in the family might raise feelings of moral responsibility, regardless of the local blood collection regime or other contextual differences between countries.

However, we also found some differences between the study results, mainly related to donor sample compositions and the magnitude of effect sizes. Regarding contextual differences between Denmark and the Netherlands, donor samples might differ as a result of organizational variation in blood collection regimes. Following Healy's categorization 7, blood collection in the Netherlands originates from a Red Cross regime which is rooted in voluntary, religious organizations, therefore being more likely to attract fewer but more loyal donors. The Danish collection regime might be more effective in recruiting new, young donors but less so in retaining them over time. This could explain why the Danish sample shows a higher proportion of younger, lapsed donors with a lower number of previous donations. Moreover, differences might be explained by BCA recruitment strategies. In the Netherlands, recruitment was shown to be related to donor diversity and loyalty 22. Differences between Danish and Dutch recruitment and retention strategies might therefore lead to different donor pools in terms of diversity and loyalty, subsequently influencing the extent to which life events impact on donor lapse.

Contextual differences other than those exerted by BCAs might contribute to differences in effect sizes. For instance, regulations regarding parental care after childbirth differ significantly between countries. In the Netherlands, fathers have only two days off after childbirth 23, posing serious constraints on their available time, subsequently increasing their lapsing risk 1. The association between childbirth and lapse is smaller for Danish donors, possibly explained by extended parental leave providing parents, especially fathers, with more time after childbirth 24. Yet, the list of explanatory contextual factors is inconclusive. For instance, starting a job showed a strong positive association with donor lapse in the Netherlands, but not in Denmark. We speculated that differences might be the result of country variations in commuting distances, importance of the work‐life balance or blood collection drives at businesses, but no such differences were found 25, 26, 27, 28. Are there contextual factors at play here, or are the different result the mere effect of data differences?

Regarding data differences, donor samples and effect sizes could differ as a result of the sole use of register data in the current study as opposed to the combination of register and survey data in the study from the Netherlands. The current study includes all active donors, while the previous study only included donors participating in both waves of the survey. Analyses showed non‐responders being more likely younger, male, lapsed donors having made a lower number of average donations, possibly explaining why the Danish sample shows a higher proportion of donors with these characteristics. Moreover, register data eliminates the possibility of introducing recall bias and the telescoping effect 29. In studying self‐reporting on the occurrence of life events in surveys, respondents more likely recall life events closely related to their donation decision (i.e. recall bias), or wrongly assign the occurrence of a life event to a specific time‐frame when this time‐frame is introduced (i.e. telescoping effect), hereby overestimating effect sizes. Register data do not include false negatives, therefore being more accurate in estimating the true effect sizes.

### Strengths and limitations

Although this study provides valuable insights on cross‐country variations in blood donor behaviour, providing more accurate and reliable estimations, our study also has some limitations. Registries typically provide more accurate and complete sources of data, but do not include relevant variables related to subjective perceptions and other individual factors such as the perceived difficulty to plan a donation and talking to other donors. Linking population‐wide registries to results from motivational questionnaires for a subset of the donor population would allow for an even better understanding of donation decisions across the blood donor career, for example understanding interrelationships between various health‐related events and their differences between countries. For now, we used variables serving as proxies for these missing variables, corresponding to other mediators from the Dutch theoretical framework: weekly working hours and the extent to which donors know other blood donors. In this way, we have used the data without introducing too many incongruences.

While using different contexts and data in replication studies, it is difficult to pinpoint exactly which differences explain inconsistencies between study results. We acknowledge this limitation. Our aim was to examine the association between life events and donor behaviour in Denmark. The comparison with the Netherlands shows that contextual and data difference could yield various study outcomes. Researchers as well as BCAs need to be aware of such differences and its implications in interpreting international study results.

### Future theoretical and practical directions

The influence of life events on blood donor behaviour across the life course so far has been studied in the Netherlands, Germany and Denmark. While certainly there are differences between these countries, they also are quite comparable with respect to collection systems as well as socio‐economic circumstances and cultural orientation 25, 26, 30, 31. It is worthwhile to further examine cross‐country differences in a broader variety of countries, including the United States and Australia, as well as African and Asian countries as to allow for conclusions on the role of contextual factors in donor behaviour across the donor career.

We suggest future studies to focus on underlying mechanisms explaining the relation between life events and donor behaviour. While the current study and the study from the Netherlands showed social and practical concerns to partially explain why life events impact on donor lapse, the bulk of these associations is still unaccounted for. For instance, neither of the two studies found evidence for social mechanisms playing a role in the relation between health‐related events and donor lapse, nor could we conclude on the different results for starting a job and donor lapse.

Further understanding of underlying mechanisms is especially important since the occurrence of specific life events that affect donors' personal resources are increasing (e.g. higher number of labour market transitions due to the rise of temporary contracts) 32. Increasing fluctuations of personal resources might affect donation decisions across the blood donor career. Retaining these donors is important as it is more cost‐effective than recruiting new donors as experienced donors are more likely to donate again, have lower no‐show rates, and guarantee safer blood compared to novice donors 33.

In‐depth studies of these associations would therefore be of practical interest in making evidence‐based decisions on the development of targeted donor retention strategies. For instance, sending postcards to donors who recently got a child might increase engagement between donors and blood banks. Promoting personal donation motivations (e.g. awareness of need, feelings of moral responsibility) might subsequently increase return rates in donors with heightened lapsing risks. Implementing targeted retention strategies require higher levels of personal contact between donors and blood banks. This will become more feasible in the coming years when services such as online donor portals become more easily available, creating opportunities for more intensive information‐sharing, subsequently increasing opportunities for segmented blood donor management.

Moreover, exploring work‐home‐donation distances might increase understanding in why people change their donation decision after starting or losing a job, and assist BCAs in deciding on where and when to open their donation locations. Strategically, positioning of collection sites might be effective in recruiting underrepresented groups of young, male and ethnically diverse donors, which is essential in maintaining a sufficient and matching blood supply 34. At the same time, BCAs need to be careful in implementing international practices to their own donor management policies since we showed that blood donors and their behaviour may differ between countries.

## Conflict of interest

The authors declare that they have no conflicts of interest relevant to the manuscript submitted to Vox Sanguinis.

## Author contributions

TP, EM, RB, WK and HU contributed to the study design. TP developed the main conceptual ideas and wrote the manuscript with support from all other authors. TP wrote the code and performed the statistical analyses, based on previous work from SA and with additional analyses by Andreas Stribolt Rigas (Copenhagen University Hospital, Rigshospitalet). All authors provided critical feedback on the interpretation of the outcomes and the final version of the manuscript.

## Appendix A A Comparison of Dutch and Danish Variables

**Table A1:** Overview of the variables used in the original Dutch study and the corresponding variables used in the current Danish replication study

Variable	The Netherlands			Denmark		
	Coding	Note	Data^a^	Coding	Note^b^	Data^a^
Blood donor lapse	0 = donation 24 months after DIS‐II 1 = no donation 24 months after DIS‐II	‐	eProgesa	0 = donation in 2013–2014 1 = no donation in 2013–2014	‐	SCANDAT
Transfusion	0 = no transfusion DIS‐I ‐ DIS‐II 1 = transfusion DIS‐I ‐ DIS‐II	Transfusion for parents, siblings or children	DIS‐I & ‐II	0 = no transfusion 2009–2012 1 = transfusion 2009–2012	‐	SCANDAT
Serious disease	0 = no disease DIS‐I ‐ DIS‐II 1 = disease DIS‐I ‐ DIS‐II	Cancer, stroke or heart attack for parents, siblings or children	DIS‐I & ‐II	0 = no serious disease 2009–2012 1 = serious disease 2009–2012	‐	LPRDIAG
Death	0 = no death DIS‐I ‐ DIS‐II 1 = death DIS‐I ‐ DIS‐II	Death of parents, siblings or children	DIS‐I & ‐II	0 = no death 2009–2012 1 = death 2009–2012	‐	DODSAARS
Childbirth	0 = no childbirth DIS‐I ‐ DIS‐II 1 = childbirth DIS‐I ‐ DIS‐II	Men aged 55 and younger and women aged 45 and younger were included	DIS‐I & ‐II	0 = no childbirth 2009–2012 1 = childbirth 2009–2012	‐	FAM
Starting a job	0 = unemployed DIS‐I ‐ DIS‐II 1 = started job DIS‐I ‐ DIS‐II	Donors who were unemployed at time of DIS‐I were included	DIS‐I & ‐II	0 = unemployed 2009–2012 1 = started job 2009–2012	‐	IDAP
Losing a job	0 = employed DIS‐I ‐ DIS‐II 1 = lost job DIS‐I ‐ DIS‐II	Donors who had a job at the time of DIS‐I were included	DIS‐I & ‐II	0 = employed 2009–2012 1 = lost job 2009–2012	‐	IDAP
Working hours	x = working hours per week	‐	DIS‐II	x = working hours per week	‐	AKM
Know other donors	0 = does not know other donors 1 = knows other donors	Survey options: friends, family, acquaintances	DIS‐II	0 = no other donors in the family 1 = other donors in the family	Only the donors in the family, not acquaintances, could be identified (i.e., spouse, child, sibling, parent)	SCANDAT
Age	x = age	‐	eProgesa	x = age	‐	BEF
Female	0 = male 1 = female	‐	eProgesa	0 = male 1 = female	‐	BEF
Educational level	1 = low 2 = middle 3 = high	1 = none, prevocational secondary, lower general secondary 2 = senior secondary vocational, senior general secondary, pre‐university 3 = higher professional, university	DIS‐I	1 = low 2 = middle 3 = high	Not the same in DK, but the categories are based on the ISCED2011[Link] to make the same categories as in Dutch data	UDDA
Number of donations	x = blood donations before DIS‐I	‐	eProgesa	x = blood donations before 2009	‐	SCANDAT

a More information on the specific datasets used to retrieve information on the variable can be found by clicking on the name of the dataset (digital view only).

b A dash (‐) indicates that the same coding was used in Danish data as in the Dutch data. When there are differences between the coding in the Danish data and the Dutch data we have added a short explanation.

International Standard Classification of Education adopted by the United Nations Educational, Scientific and Cultural Organization (UNESCO) in 2011 to be able to make international comparisons with regard to educational systems.

## Appendix B Results from the Netherlands

**Table A2:** Descriptive statistics of study measures, for the total blood donor sample in the Netherlands and stratified by donor status^a^^,^ b

Characteristic	All donors (*n* = 20 560)	Active donors (*n* = 15 363; 74·7%)	Lapsed donors (*n* = 5197; 25·3%)
Life events			
Transfusion	1,855 (9·0%)	1,425 (9·3%)	430 (8·3%)
Serious disease	8,319 (40·5%)	6,206 (40·4%)	2,113 (40·7%)
Death	3,884 (18·9%)	2,966 (19·3%)	918 (17·7%)
Childbirth	2,071 (10·1%)	1,268 (8·3%)	803 (15·5%)
Starting a job	469 (2·3%)	343 (2·2%)	126 (2·4%)
Losing a job	622 (3·0%)	447 (2·9%)	175 (3·4%)
Costs			
Working hours	25·6 (±16·7)	26·7 (±16·4)	22·5 (±17·3)
Social network			
Know other donors	15,056 (73·2%)	11,481 (74·7%)	3,575 (68·8%)
Age	46·7 (±12·28)	46·7 (±11·38)	46·9 (±14·61)
Sex			
Male	9,706 (47·2%)	7,606 (49·5%)	2,100 (40·5%)
Female	10,854 (52·8%)	7,757 (50·5%)	3,097 (59·6%)
Educational level			
Low	517 (2·5%)	363 (2·4%)	154 (3·0%)
Middle	12,479 (60·7%)	9,513 (61·9%)	2,966 (57·1%)
High	7,476 (36·4%)	5,417 (35·3%)	2,059 (39·6%)
Previous blood donations	21 (10–37)	23 (11–39)	16 (8–31)

a Data reported as mean (±SD), number (%), or median (25^th^–75^th^).

b Adapted from the study by Piersma and colleagues.^1^

**Table A3:** Results for the logistic regression analyses of life events on blood donor lapse in the Netherlands^a^^,^ b mediated by the hypothesized mechanisms^c^

Life events & mechanisms^d^	Model A			Model B			*Z*_*Mediation*_	
	B^e^	SE	OR^f^ (95% CI)	B	SE	OR (95% CI)	% mediated^g^	*Z*‐value
Transfusion	−0·13*	0·06	0·87 (0·78–0·98)	−0·14*	0·06	0·87 (0·79–0·98)		
Know other donors				−0·28***	0·04	0·76 (0·70–0·81)	‐	1·33
Serious disease	0·02	0·03	1·02 (0·95–1·09)					
Death	−0·11*	0·04	0·90 (0·83–0·98)	−0·12*	0·04	0·89 (0·82–0·97)		
Know other donors				−0·27***	0·04	0·77 (0·71–0·83)	‐	0·15
Childbirth	0·60***	0·06	1·83 (1·63–2·00)	0·49***	0·06	1·64 (1·46–1·84)		
Start job	0·30***	0·15	1·34 (1·02–1·77)	0·12	0·23	1·08 (0·77–1·38)		
Lose job	0·40***	0·09	1·50 (1·25–1·80)	0·39***	0·10	1·48 (1·48–1·23)		
Know other donors				–0·27***	0·05	0·76 (0·70–0·84)	3%	2·68**

a Blood donor lapse for non‐medical reasons.

b Adapted from the study by Piersma and colleagues.^1^

c Effects are estimated separately for each life event and its hypothesized mechanisms, and only when the donor is at risk for experiencing the life event: transfusion, disease and death (*n* = 20 560), childbirth (*n* = 11 695), start job (*n* = 1713) and lose job (*n* = 15 356).

d Results adjusted for donors’ sex, age, educational level, religious affiliation and the total number of previous blood donations.

e Estimated unstandardized regression coefficients.

f OR indicates the likelihood for donor lapse compared with the reference category.

g Percentage reported only when all paths in the model were significant.^15^

****P *<* *0·001, ***P *<* *0·01, **P *<* *0·05 (two‐tailed tests).

**Table A4:** Results for the stepwise regression analyses^a^ of the health‐related life events on donor lapse^b^ in the Netherlands^c^

Life events^d^	Model 1		Model 2		Model 3		Model 4		Model 5		Model 6		Model 7	
	B^e^	SE	B	SE	B	SE	B	SE	B	SE	B	SE	B	SE
Transfusion	−0·138*	0·058					−0·150*	0·059	−0·127*	0·060			−0·142*	0·061
Serious disease			0·013	0·034			−0·022	0·035			−0·041	0·036	0·047	0·037
Death					−0·109*	0·043			−0·091*	0·044	−0·120**	0·045	−0·112*	0·045

a Effects are estimated separately for each health‐related life event in Models 1–3, in combination with one of the other health‐related life events in Models 4–6, simultaneously for all three health‐related life events in Model 7, and only when the donor is at risk for experiencing the event (*n* = 20 560).

b Blood donor lapse for non‐medical reasons.

c Data adapted from the study by Piersma and colleagues.^1^

d Results adjusted for donors’ sex, age, educational level and the total number of previous blood donations.

e Estimated unstandardized regression coefficients.

****P *<* *0·001, ***P *<* *0·01, **P *<* *0·05 (two‐tailed tests).

## Appendix C Correlation Matrix

**Table A5:** Correlation matrix including Spearman correlations (*r*) between all study measures

Measure^a^	1	2	3	4	5	6	7	8	9	10	11	12	13
1. Blood donor lapse	–												
2. Transfusion	−0·02***	–											
3. Serious disease	−0·03***	0·30***	–										
4. Death	−0·03***	0·35***	0·35***	–									
5. Childbirth	0·04***	−0·02***	−0·01***	−0·05***	–								
6. Starting a job	−0·01	−0·01	−0·01	−0·00	0·08***	–							
7. Losing a job	0·05***	−0·02***	−0·02***	−0·02***	−0·00	0·00	–						
8. Working hours	−0·08***	0·04***	0·07***	0·05***	0·03***	0·29***	−0·28***	–					
9. Know other donors	−0·08***	−0·00	0·00	−0·01**	0·04***	0·05***	−0·00	0·44***	–				
10. Age	−0·03***	0·04***	−0·01***	0·09***	−0·33***	−0·00	−0·15***	0·07***	0·03***	–			
11. Sex	0·07***	−0·01*	−0·01*	−0·02***	−0·00	0·02	0·03***	0·02***	0·01*	−0·09***	–		
12. Education	−0·03***	0·00	0·03***	0·00	0·12***	0·18***	−0·11***	0·18***	−0·00	0·03***	0·04***	–	
13. Previous donations	−0·12***	0·03***	0·03***	0·06***	−0·09***	0·06***	−0·11***	−0·12***	−0·13***	0·04***	0·07***	0·07***	–

a Measures ordered by category: blood donor lapse (1), life events (2–7), mechanisms (8–9), and control variables (10–13).

****P *<* *0·001, ***P *<* *0·01, **P *<* *0·05 (two‐tailed tests).
